# Analysis of microbiological tests in patients withholding or withdrawing life-sustaining treatment at the end stage of life in 2 Korean hospitals

**DOI:** 10.1017/ice.2023.191

**Published:** 2024-02

**Authors:** Sohyun Bae, Ki Tae Kwon, Soyoon Hwang, Yoonjung Kim, Hyun-Ha Chang, Shin-Woo Kim, Nan Young Lee, Yu Kyoung Kim, Je Chul Lee

**Affiliations:** 1 Division of Infectious Diseases, Department of Internal Medicine, Kyungpook National University Hospital, School of Medicine, Kyungpook National University, Daegu, Republic of Korea; 2 Division of Infectious Diseases, Department of Internal Medicine, Kyungpook National University Chilgok Hospital, School of Medicine, Kyungpook National University, Daegu, Republic of Korea; 3 Department of Clinical Pathology, School of Medicine, Kyungpook National University, Daegu, Republic of Korea; 4 Department of Microbiology, School of Medicine, Kyungpook National University, Daegu, Republic of Korea

## Abstract

**Objective:**

We evaluated the adequacy of microbiological tests in patients withholding or withdrawing life-sustaining treatment (WLST) at the end stage of life.

**Setting:**

The study was conducted at 2 tertiary-care referral hospitals in Daegu, Republic of Korea.

**Design:**

Retrospective cross-sectional study.

**Methods:**

Demographic findings, clinical and epidemiological characteristics, statistics of microbiological tests, and microbial species isolated from patients within 2 weeks before death were collected in 2 tertiary-care referral hospitals from January to December 2018. We also reviewed the antimicrobial treatment that was given within 3 days of microbiological testing in patients on WLST.

**Results:**

Of the 1,187 hospitalized patients included, 905 patients (76.2%) had WLST. The number of tests per 1,000 patient days was higher after WLST than before WLST (242.0 vs 202.4). Among the category of microbiological tests, blood cultures were performed most frequently, and their numbers per 1,000 patient days before and after WLST were 95.9 and 99.0, respectively. The positive rates of blood culture before and after WLST were 17.2% and 18.0%, respectively. *Candida* spp. were the most common microbiological species in sputum (17.4%) and urine (48.2%), and *Acinetobacter* spp. were the most common in blood culture (17.3%). After WLST determination, 70.5% of microbiological tests did not lead to a change in antibiotic use.

**Conclusions:**

Many unnecessary microbiological tests are being performed in patients with WLST within 2 weeks of death. Microbiological testing should be performed carefully and in accordance with the patient’s treatment goals.

Many patients at the end stage of life (ESOL) have received substantial antibiotic therapy due to their vulnerability to microbial infections.^1,2^ In Korea, the Hospice and Palliative Care and Dying Patients’ Decisions on Life-Sustaining Treatment for Patients at the End of Life Act (Life-Sustaining Treatment Decision Act, LSTDA) was enacted in February 2016.^3^ However, antibiotic therapy has never been publicly discussed in the decision-making framework for patients at ESOL as much as other life-sustaining treatment decisions, such as cardiopulmonary resuscitation, ventilator therapy, hemodialysis, anticancer chemotherapy, extracorporeal life support, transfusion, and inotropic treatments.^4,5^ According to a recent nationwide study in South Korea, 88.9% of 1,201 patients received a median of 2 antimicrobial agents within 2 weeks before death.^6^ In a 2-center study of 1,296 inpatients on withholding or withdrawing life-sustaining treatment (WLST), 90.6% of those who died received antibiotic therapy within 7 days before death.^7^

Because antibiotics are often used in patients at ESOL and microbiological tests such as blood cultures are commonly performed before antibiotic therapy, terminally ill patients undergo substantial microbiological testing. Excessive microbiological testing could increase the psychological and financial burden, and false-positive results could lead to misinterpretation.^8,9^ To date, the extent of microbiological testing in ESOL has never been investigated. Few studies have investigated the types of microorganisms in ESOL.

In this study, we aimed to identify the burden of microbiological tests and to evaluate their impact on antibiotic use in patients on WLST at the ESOL within the 2 weeks before death.

## Methods

### Study design and data collection

This retrospective cross-sectional study was conducted at 2 tertiary-care referral hospitals in Korea between January and December 2018. This study was based on a chart review of patients who died in the hospital during the study period. Retrospective data (ie, demographic findings, clinical and epidemiological characteristics, microbiological tests, and microbial isolates) were collected from patients within 2 weeks before death. The duration of antibiotic therapy for common infectious diseases, including sepsis, is generally 2 weeks, and microbiological tests are often performed before antibiotics are started. Therefore, we assumed that the maximum duration of antibiotic use before death was 2 weeks, and we reasoned that microbiological tests performed within 2 weeks before death could be representative of the microbiological testing in patients at the ESOL. Previous studies have also collected data within 2 weeks before death when investigating clinical characteristics or antibiotics in patients close to death, such as those with terminal illnesses.^10,11^

Specimens included blood, urine, sputum, ascites, or any site discharge from patients aged over 18 years. Patients who died within 48 hours of admission were excluded. We extracted the microbiological test statistics from a computerized hospital information system. The same type of specimen was counted once a day. If different types of specimens were collected, they were counted separately, even on the same day. If the same type of specimen was tested on different days, it was counted separately. We do not usually repeat the same type of microbiological test on the same day. To evaluate the necessity of microbiological tests, we also reviewed the antimicrobial treatment that was given within 3 days after microbiological tests performed in patients on WLST. Because it takes an average of 3 days to obtain the results of microbiological tests, we looked at changes in antibiotic treatment over a 3-day period.^12^

Given the retrospective nature of the study and the use of anonymous clinical data for analysis, the Institutional Review Board of Kyungpook National University Hospital (IRB no. KNUH 2019-06-011) waived the ethical review of this study and the requirement for informed consent on June 7, 2019.

### Definition

In Korea, the LSTDA was enacted in February 2016 and was fully implemented in February 2018 after a trial period. According to the LSTDA, Patients at the ESOL are legally defined as those who have received a medical assessment from the doctor in charge and 1 medical specialist in the relevant field to be in a state of imminent death, in which there is no possibility of revitalization or recovery despite treatment, and in whom symptoms worsen rapidly.^5^ Life-sustaining treatment is defined as medical treatment by cardiopulmonary resuscitation, hemodialysis, administering anticancer drugs, mechanical ventilation, and other medical treatments prescribed by Presidential Decree to a patient at the ESOL, which merely extends the duration of the ESOL without curative effects.^5^ WLST is defined as not performing life-sustaining treatment for the patient at the ESOL from the beginning.^5^ WLST is defined as discontinuing life-sustaining treatment for the patient at the ESOL who has been receiving the treatment.^5^ WLST patients are defined as WLST group and the patients continuing life-sustaining treatment (CLST) are defined as CLST group. Hospice and palliative care is defined as medical care provided to a terminal patient or patient at the ESOL and his or her family for the purpose of comprehensively evaluating and providing treatment in physical, psychosocial, and spiritual domains, including pain and symptom relief.^5^ Terminal patients are defined as those who have been diagnosed as expected to die within a few months by the doctor in charge and 1 medical specialist in the relevant field because there is no possibility of a fundamental recovery, and their symptoms are gradually worsening despite proactive treatment.^5^ Comorbidities of included patients were collected from a death certificate in the electronic medical record.

### Microbiological identification and molecular analysis

All species and antimicrobial susceptibilities were identified using Microscan (Siemens Healthcare Diagnostics, CA, USA) or VITEK II (bioMerieux, Marcy l’Etoile, France), according to the guidelines of the Clinical Laboratory Standards Institute.^13^

### Statistical analyses

Categorical variables have been expressed as numbers and percentages and have been compared using the χ^2^ or the Fisher exact test. Continuous variables have been expressed as mean ± standard deviation (SD) or median with interquartile range (IQR) values and have been compared using the Student *t* test or the Mann–Whitney *U* test. All statistical data were analyzed using R statistics version 3.1 software (R Foundation for Statistical Computing, Vienna, Austria). *P* values < .05 were considered significant.

## Results

### Demographic features and clinical characteristics of the patients

In total, 1,187 patients died during their hospitalization and were included in the study. Of these, 905 patients (76.2%) were determined to have had WLST. Table 1 shows the demographic features and clinical characteristics of the 1,187 patients included: 789 (66.5%) were men and 398 (33.5%) were women, and the mean age was 68.6 ± 13.5 years. The mean length of hospital stay was 20.5±23.8 days. The proportion of patients with an infectious episode identified in the 2 weeks before death was 24.8%, and respiratory infection was most common (17.7%), followed by urinary tract infection (1.4%) and skin and soft-tissue infection (1.1%). Solid tumors (54.7%) were the most common comorbidity, followed by diabetes mellitus (19.1%), neurologic disease (14.1%), and cardiovascular disease (9.4%).

**Table 1.** Comparison of Demographic Features and Clinical Characteristics Between WLST and CLST Group

Variable	Total (N = 1,187), No. (%)^a^	WLST Group (N = 905), No. (%)^a^	CLST group (N = 282), No. (%)^a^	*P* Value
Age, y ±SD)	68.6 ± 13.5	69.1 ± 13.2	67.1 ± 14.6	.045
Sex, female	398 (33.5)	311 (34.4)	87 (30.9)	.308
LOS, median days ( ± SD)	20.5 ± 23.8	20.9 ± 24.3	19.1 ± 22.1	.272
**Infectious episode**	294 (24.8)	224 (24.8)	70 (24.8)	1.000
Respiratory	210 (17.7)	165 (18.2)	45 (16.0)	.433
Urinary	16 (1.4)	9 (1.0)	7 (2.5)	.110
Skin and soft tissue	13 (1.1)	7 (0.8)	6 (2.1)	.114
Others	61 (5.1)			
**Comorbidity**				
Solid tumor	649 (54.7)	551 (60.9)	98 (34.8)	<.001
Hematologic malignancy	80 (6.7)	52 (5.7)	28 (9.9)	.021
Neurologic disease	167 (14.1)	113 (12.5)	54 (19.1)	.007
Spinal injury	7 (0.6)	6 (0.7)	1 (0.4)	.885
Diabetes mellitus	227 (19.1)	168 (18.6)	59 (20.9)	.428
Chronic lung disease	74 (6.2)	58 (6.4)	16 (5.7)	.761
Cardiovascular disease	111 (9.4)	75 (8.3)	36 (12.8)	.032
Chronic kidney disease	46 (3.9)	26 (2.9)	20 (7.1)	.002
End-stage renal disease	25 (2.1)	9 (1.0)	16 (5.7)	<.001
Chronic liver disease	53 (4.5)	37 (4.1)	16 (5.7)	.337

Note. WLST, withholding or withdrawing life-sustaining treatment; CLST, continuing life-sustaining treatment.

a Units unless otherwise specified.

Comparing the comorbidities in the WLST and CLST groups, solid tumors were more common in the WLST group (60.9% vs 34.8%; *P* < .001), and hematological malignancies (5.7% vs 9.9%; *P* = .021), cardiovascular disease (8.3% vs 12.8%; *P* = .032), chronic kidney disease (2.9% vs 7.1%; *P* = .002), and end-stage renal disease (1.0% vs 5.7%; *P* < .001) were more common in the CLST group.

### Statistics of microbiological tests within 2 weeks before death

Table 2 shows the statistics on microbiological tests in the CLST group versus the WLST group in the 2 weeks before death. The CLST group underwent a greater number of tests per 1,000 patient days than WLST group (288.0 vs 216.6). Blood cultures were the most frequently performed test within 2 weeks before death: 2.7 and 2.0 times in the CLST and WLST groups, respectively. For the WLST group, statistics on microbiological tests before and after WLST determination are shown in Table 3. The number of tests per 1,000 patient days was higher after WLST than before WLST (242.0 vs 202.4). Blood culture was the most frequently performed test: 1.8 and 1.5 times before and after WLST, respectively. The positive rates of blood cultures before and after WLST were 17.2% and 18.0%, respectively.

**Table 2.** Statistics of Microbiological Tests in Patients Continuing Versus Withholding or Withdrawing Life-Sustaining Treatment Within 2 Weeks Before Death (CLST Group vs WLST Group)

	Total Number of Patients Within 2 Weeks Before Death (N = 1,187)									
Category	CLST Group (N = 282)					WLST Group (N = 905)				
	Patients, No. (%)	Tests, No. (%)^a^	Tests per 1,000 Patient Days	Positive Results, No. (%)^b^	Tests per Patient, No.	Patients, No. (%)	Tests, No. (%)^a^	Tests per 1,000 Patient Days, No.	Positive Results, No. (%)^b^	Tests per Patient, No.
Blood culture	205 (72.7)	548 (48.2)	138.8	137 (25.0)	2.7	608 (97.0)	1229 (44.8)	97.0	215 (17.5)	2.0
Sputum culture	123 (43.6)	235 (20.7)	59.5	233 (99.1)	1.9	324 (35.8)	572 (20.8)	45.1	565 (98.8)	1.8
Urine culture	107 (37.9)	181 (15.9)	45.8	58(32.0)	1.7	323 (35.7)	526 (19.2)	41.5	229 (43.5)	1.6
Other culture	83 (29.4)	132 (11.6)	34.4	51 (38.6)	1.6	198 (21.9)	302 (11.0)	23.8	111 (36.8)	1.5
*C. diff* ELISA	36 (12.8)	40 (3.5)	10.1	4 (10.0)	1.1	96 (10.6)	105 (3.8)	8.3	15 (14.3)	1.1
*C. diff* PCR	0 (0.0)	0 (0.0)	0.0	0 (0.0)	0.0	6 (1.0)	10 (0.4)	0.8	1 (10.0)	1.1
Any test	232 (82.3)	1,137 (100.0)	288.0	483 (38.5)	4.9	686 (75.8)	2,744(100.0)	216.6	1,136 (41.4)	4.0

Note. CLST, continuing life-sustaining treatment; WLST, withholding or withdrawing life-sustaining treatment; *C, diff, Clostridioides difficile*; ELISA, enzyme-linked immunosorbent assay; PCR, polymerase chain reaction.

a No. of tests per patient among patients undergoing microbiological testing during study periods (no. of tests/no. of patients).

b Positivity rates per test.

**Table 3.** Statistics on Microbiological Tests Performed Before and After Withholding or Withdrawing Life-Sustaining Treatment (WLST) Within 2 Weeks Before Death

	Total No. of Patients With WLST (N = 905) Within 2 Weeks Before Death									
Category	Before WLST determination					After WLST Determination				
	Patients, No. (%)	Tests, No. (%)^a^	Tests per 1,000 Patient Days	Positive Results, No. (%)^b^	Tests per Patient, No.	Patients, No. (%)	Tests, No. (%)^a^	Tests per 1,000 Patient Days, No.	Positive Results, No. (%)^b^	Tests per Patient, No.
Blood culture	441 (48.7)	780 (47.4)	95.9	134 (17.2)	1.8	297 (32.8)	449 (40.9)	99.0	81 (18.0)	1.5
Sputum culture	199 (22.0)	312 (19.0)	38.4	307 (98.4)	1.6	175 (19.3)	260 (23.7)	57.3	258 (99.2)	1.5
Urine culture	196 (21.7)	293 (17.8)	36.0	119 (40.6)	1.5	162 (17.9)	233 (21.2)	51.4	110 (47.2)	1.4
Other culture	136 (15.0)	191 (11.6)	23.5	67 (35.1)	1.4	75 (8.3)	111 (10.1)	24.5	44 (39.6)	1.5
*C. difficile* ELISA	55 (6.1)	61 (3.7)	7.5	10 (11.1)	1.1	41 (4.5)	44 (4.0)	9.7	5 (11.4)	1.1
*C. difficile* PCR	8 (0.9)	9 (0.5)	1.1	1 (18.8)	1.1	1 (0.1)	1 (0.1)	0.2	0 (0)	1.0
Any test	**502 (55.5)**	1,646 (100.0)	202.4	638 (38.8)	3.3	**405 (44.9)**	1,098 (100.0)	242.0	498 (45.4)	2.7

Note.WLST, withholding or withdrawing life-sustaining treatment; *C difficile*, *Clostridioides difficile*; ELISA, enzyme-linked immunosorbent assay; PCR, polymerase chain reaction.

a No. of tests per patient among patients undergoing microbiological testing during study periods (no. of tests/no. of patients).

b Positivity rates per test.

### Distribution of microbial species within 2 weeks before death

**Figure 1.**
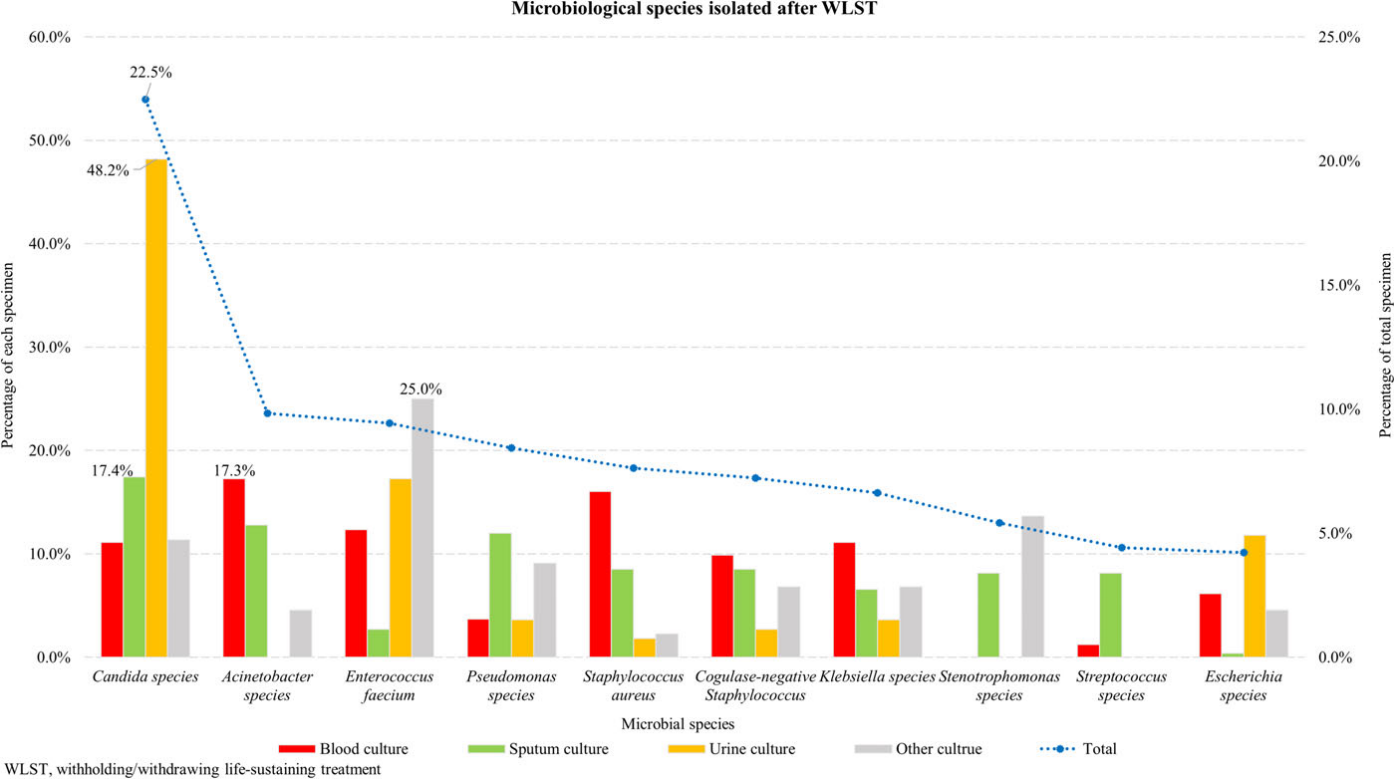
Microbiological species isolated in 905 patients after WLST within 2 weeks before death. The blue dots represent the percentage of total strains and are connected by a dotted line. They are shown from left to right in the order of the most frequent species. *Candida* spp. were the most common, followed by *Acinetobacter* spp., and then *Enterococcus faecium*. Red bars represent blood cultures; green bars represent sputum cultures; yellow bars represent urine cultures; and grey bars represent other cultures. The height of the bars indicates the percentage of each specimen.

Figure 1 shows the distribution of microbial species isolated after determination of WLST in 905 patients. *Candida* spp. (22.5%) were the most common, followed by *Acinetobacter* spp. (9.8%), *Enterococcus faecium* (9.4%), *Pseudomonas* spp. (8.4%), *Staphylococcus aureus* (7.6%), coagulase-negative *staphylococci* (7.2%), *Klebsiella* spp. (6.6%), *Stenotrophomonas* spp. (5.4%), *Streptococcus* spp. (4.4%), and *Escherichia* spp. (4.2%). *Candida* spp. were the most common microbiological species in sputum (17.4%) and urine (48.2%), and *Acinetobacter* spp. were the most common in blood culture (17.3%).

### Change in antibiotic regimen based on microbiological testing in patients on WLST

Table 4 shows the change in antibiotic regimen based on microbiological tests performed in patients on WLST. Of 1,098 tests, 774 tests (70.5%) did not lead to any change in antibiotic use, and 25 tests (2.3%) did not result in antibiotic use after microbiological testing. In the 749 tests with no change in antibiotic regimen, a combination of carbapenem and glycopeptide (163 tests, 21.8%) was the most common antibiotic regimen used prior to testing. Of the 324 tests (29.5%) that led to an antibiotic change, starting new antibiotics was the most common at 118 tests (10.7%), followed by switching from a noncarbapenem to a carbapenem at 89 tests (8.1%).

**Table 4.** Changes in Selected Antibiotics Within 3 Days After Microbiological Testing on Withholding or Withdrawing Life-Sustaining Treatment (WLST Group)

Changes in Selected Antibiotics	Tests (N = 1,098), No. (%)
**No**	774 (70.5)
No use of antibiotics	25 (2.3)
No change in antibiotics	749 (68.2)
**Yes**	324 (29.5)
Starting an antibiotic use	118 (10.7)
Adding other antibiotics	33 (3.0)
Switch from noncarbapenem to carbapenem	89 (8.1)
Switch from noncarbapenem to noncarbapenem	44 (4.0)
Switch from carbapenem to noncarbapenem	22 (2.0)
Switch from carbapenem to another carbapenem	4 (0.4)
Stopping antibiotic use	14 (1.3)

## Discussion

Few studies have briefly referred to microbiological cultures before initiating antibiotic treatment in patients at the ESOL. Antimicrobial stewardship research on the ESOL has focused on the use of antibiotics, with less attention to the burden of microbiological testing and impact of test results on antibiotic use. It is necessary to understand the current situations for microbiological testing and results in patients at the ESOL to provide proper care and to reduce unnecessary antimicrobial therapy.

A Korean study of changes in life-sustaining treatment after do-not-resuscitate (DNR) orders in patients with terminal cancer found that 69 (34.5%) of 200 patients received additional blood tests even after a DNR order.^14^ In Japan, a retrospective study reported that blood cultures were performed in 28 (45.2%) of 62 patients with terminal cancer, of whom 4 (14.3%) had positive results.^15^ These rates are similar to the 32.8% of patients who received blood cultures after WLST in this study. In the aforementioned Japanese study, researchers mentioned that 90% of terminally ill cancer patients did not experience symptom improvement despite antibiotic treatment after blood culture.^15^ A previous study of 3,890 blood cultures showed that blood cultures were inefficient at detecting bacteremia, with 13.9% positive results and a low yield of 7.5% after correcting for contamination.^16^ According to the LSTDA, the purpose of life-sustaining treatment for patients at the ESOL is to protect the dignity and value of human beings by assuring the best interests of the patients and by respecting their self-determination.^17^ Sometimes, clinicians may reflexively order microbiological tests.^18^ However, low yields and high rates of false-positive blood cultures can lead to unnecessary antibiotics such as vancomycin, which can cause nephrotoxicity, additional tests, unnecessary removal of venous catheters, longer hospital stays, and increased costs.^19,20^ Numerous microbiological tests can have adverse effects including patient discomfort (phlebitis from blood cultures, pain from testing, etc.) and psychological anxiety.

*Candida* spp. (22.5%) were the most common isolates, followed by *Acinetobacter* spp. (9.5%). *Candida* spp. were the most common isolates in sputum and urine cultures. *Candida* spp. are commensals found in the human mouth, skin, upper respiratory tract, and intestine.^21,22^ Low-quality sputum specimens can confuse antibiotic use due to contamination and inappropriate sampling.^23^ Clinicians sometimes prescribe antimicrobials even if they get a false-positive result because they are afraid that the infection will get worse.^8,24–26^ We have found that many of the microbiological results isolated after the determination of WLST can be false positives, representing contaminants or colonizers. In blood cultures, *Acinetobacter* spp. were the most common isolates, followed by *Staphylococcus aureus* and *Enterococcus faecium*, which are considered true pathogens. In patients who have WLST, we believe that bloodstream infections with these species are part of the disease process leading to death and need not be treated as sepsis.

Typically, clinicians treat empirically with broad-spectrum antibiotics until a microbiological cause can be identified by culture or other tests. They then switch to the narrowest spectrum antibiotic once culture and susceptibility data are available.^27^ However, despite numerous tests in patients on WSLT, microbiological testing often did not lead to changes in antibiotic therapy, especially in patients on broad-spectrum antibiotics. Our results also show that the most common change was from a noncarbapenem to a carbapenem, which represents a broadening of the antibiotic spectrum. Our findings on antibiotic use suggest that a significant amount of unnecessary microbiological testing is being performed.

Our study had several limitations. First, because data were collected from only 2 centers, these results may not be generalizable. However, the 2 tertiary-care university hospitals represent the Daegu-Gyengsangbuk-do area, encompassing ∼10.0% of the Korean population. Second, this study did not include outpatient hospice patients. In South Korea, 77.1% of deaths occurred among patients admitted to medical institutions, which means that most people, especially those aged ≥65 years, died in medical institutions, including nursing homes. Given this situation, we believe that the inpatient population is sufficient to evaluate the adequacy of microbiological tests at the end stage of life.^28^ Third, in rare cases, the same type of specimen may have been collected multiple times in a same day, which may underestimate the overall microbiological tests. Fourth, comorbidities may not have been accurate because these data were extracted from death certificates rather than from medical records.

In conclusion, our study shows that numerous microbiological tests are being performed even after a WLST determination has been made. Most tests have negative results, and many of the positive results are not clinically significant. In many cases, there was no change in the use of antibiotics after the microbiological tests were performed. These findings suggest that many unnecessary microbiological tests are being performed in patients on WLST within 2 weeks before death. Unnecessary microbiological testing can cause discomfort and unnecessary treatment for patients, can increase costs, and can increase workload for the laboratory. In patients at the ESOL, not only antibiotic use but also microbiological testing should be performed carefully and in line with the patient’s treatment goals. Further prospective studies are needed on the appropriateness and usefulness of microbiological testing in patients at the ESOL.
